# Supplementary material for: Crude metabolites from endophytic fungi inhabiting Cameroonian Annona muricata inhibit the causative agents of urinary tract infections
Source: PLoS One. 2022 May 11;17(5):e0267246. doi: 10.1371/journal.pone.0267246 (PMC9094522; doi:10.1371/journal.pone.0267246)
Supplement: S1 File — (DOCX) [file pone.0267246.s001.docx]

**Crude metabolites from endophytic fungi inhabiting Cameroonian *Annona muricata* inhibit the causative agents of urinary tract infections**

Lorette Victorine Yimgang, Rufin Marie Kouipou Toghueo*, Ines Michele Kanko Mbekou, Darline Dize, Fabrice Fekam Boyom^*^

Antimicrobial & Biocontrol Agents Unit (AmBcAU), Laboratory for Phytobiochemistry and Medicinal Plants Studies, Department of Biochemistry, Faculty of Science, University of Yaoundé I, Cameroon, P.O. Box 812, Messa, Yaoundé, Cameroon;

*Correspondence: toghueo.rufin@yahoo.fr (RMKT) and fabrice.boyom@fulbrightmail.org (FFB). Antimicrobial and Biocontrol Agents Unit, Laboratory for Phytobiochemistry and Medicinal Plants Studies, Department of Biochemistry, Faculty of Science, University of Yaoundé I, P.O. Box 812, Messa, Yaoundé, Cameroon

**Table S1.** Extraction yields (mg/100mL) and screening results of extracts from endophytic fungi from various organs of *Annona muricata*

| Plant organs | Extract codes | Yield (mg) | *Escherichia coli* ATCC 25922 | *Klebsiella oxytoca* isolate | *Staphylococcus aureus* ATCC 43300 |
| --- | --- | --- | --- | --- | --- |
| Roots | AMr5 | 48 | - | - | - |
|  | AMr7 | 28 | - | - | - |
|  | AMr8 | 24 | - | - | - |
|  | AMr9 | 195 | + | + | + |
|  | AMr10 | 12 | - | - | + |
| Root bark | AMrb1 | 95 | + | + | + |
|  | AMrb9 | 258 | + | + | + |
|  | AMrb10_1_ | 150 | - | - | - |
|  | AMrb11 | 61 | + | + | + |
|  | AMrb13 | 14 | - | - | - |
|  | AMrb25 | 81 | - | - | - |
| Fruits | AMf1 | 126 | + | + | + |
|  | AMf2 | 85 | - | - | - |
|  | AMf3 | 67 | + | + | + |
|  | AMf4 | 216 | + | + | + |
|  | AMf5 | 16 | - | - | - |
|  | AMf6 | 22 | + | + | + |
| Seeds | AMs1 | 402 | - | - | - |
|  | AMs3 | 264 | + | + | + |
|  | AMs9 | 139 | + | + | + |
|  | AMs10 | 116 | - | - | - |
| Thorns of fruit | AMtf5 | 95 | + | + | + |
|  | AMtf6 | 96 | - | - | - |
|  | AMtf9 | 110 | - | - | - |
|  | AMtf12 | 100 | - | - | - |
|  | AMtf15 | 56 | + | + | + |
|  | AMtf16 | 120 | - |  |  |
| Twigs | AMtw3 | 48 | + | + | + |
|  | AMtw5 | 103 | - | - | - |
|  | AMtw7 | 32 | - | - | - |
|  | AMtw8 | 46 | - | - | - |
|  | AMtw | 80 | - | - | - |
| Leaves | AML3 | 100 | - | - | - |
|  | AML10 | 24 | - | - | - |
| Stem bark | AMsb1 | 194 | + | + | + |
|  | AMsb14 | 130 | - | - | - |
|  | AMsb16 | 130 | - | - | - |
|  | AMsb23 | 25 | + | + | + |
|  | AMsb25 | 60 | - | - | - |
| Barks | AMb7 | 24 | + | + | + |
| Peducles | AMpe9 | 121 | - | - | - |
|  | PDB | 21 | - | - | - |

The yields of extraction were expressed as mg/100 mL of culture medium after culture as described above; -: not active at 100 µg /mL; +: active at 100 µg /mL; AMr: *A. muricata* root, AMrb: *A. muricata* root bark, AMf: *A. muricata* fruit, AMs: *A. muricata* seed, AMtf: *A. muricata* thorn of fruit, AMtw: *A. muricata* twigs, AMl: *A. muricata* leaves, AMb: *A. muricata* bark, AMsb: *A. muricata* stem bark, AMpe: *A. muricata* peducle, PDB: Potato Dextrose Broth.

**File S2.** ITS sequences of the seventeen endophytic fungi selected from the antibacterial screening

> AMtf15

GCCGGGGGCTCTCANCCCCGGGCCCGCGCCCGCCGGAGACACCACGAACTCTGTCTGATCTAGTGAAGTCTGAGTTGATTGTATCGCAATCAGTTAAAACTTTCAACAATGGATCTCTTGGTTCCGGCATCGATGAAGAACGCAGCGAAATGCGATAACTAGTGTGAATTGCAGAATTCCGTGAATCATCGAGTCTTTGAACGCACATTGCGCCCCCTGGTATTCCGGGGGGCATGCCTGTCCGAGCGTCATTGCTGCCCATCAAGCACGGCTTGTGTGTTGGGTCGTCGTCCCCTCTCCGGGGGGGACGGGCCCCAAAGGCAGCGGCGGCACCGCGTCCGATCCTCGAGCGTATGGGGCTTTGTCACCCGCTCTGTAGGCCCGGCCGGCGCTTGCCGAACGCAAATCAATCTTTTCCAGGTTGACCTCGGATCAGGTAGGGATACCCGCTGAACTTAAGCATAT

> AMf6

ACGGCCCCCCTGAACGCTGTCTGAAGTTGCAGTCTGAGACCTATAACGAAATTAGTTAAAACTTTCAACAACGGATCTCTTGGTTCCGGCATCGATGAAGAACGCAGCGAAATGCGATAACTAATGTGAATTGCAGAATTCAGTGAATCATCGAGTCTTTGAACGCACATTGCGCCCTCTGGTATTCCGGAGGGCATGCCTGTCCGAGCGTCATTGCTGCCCTCAAGCCCGGCTTGTGTGTTGGGCCCCGTCCCCCCCGCCGGGGGGACGGGCCCGAAAGGCAGCGGCGGNACCGCGTCCGGTCCTCGAGCGTATGGGGCTTCGTCACCCGCTCTAGTAGGCCCGGCCGGCGCCNGCCGACCCCCAACCTTTAATTATCTCAGGTTGACCTCGGATCAGGTAGGGATACCCGCTGAACTTAAGCATAT

> AMsb1

CCTATGTTGCCTCGGCGGGCCCCGCGCCCNCCGACGGCCCCCCTGAACGCTGTCTGAAGTTGCAGTCTGAGACCTATAACGAAATTAGTTAAAACTTTCAACAACGGATCTCTTGGTTCCGGCATCGATGAAGAACGCAGCGAAATGCGATAACTAATGTGAATTGCAGAATTCAGTGAATCATCGAGTCTTTGAACGCACATTGCGCCCTCTGGTATTCCGGAGGGCATGCCTGTCCGAGCGTCATTGCTGCCCTCAAGCCCGGCTTGTGTGTTGGGCCCCGTCCCCCCCGCCGGGGGGACGGGCCCGAAAGGCAGCGGCGGCACCGCGTCCGGTCCTCGAGCGTATGGGGCTTCGTCACCCGCTCTAGTAGGCCCGGCCGGCGCCAGCCGACCCCCAACCTTTAATTATCTCAGGTTGACCTCGGATCAGGTAGGGATACCCGCTGAACTTAAGCATAT

> AMf3

AACTCTTGCTTTGGTTTGGCCTAGAAATAGGTTGGGCCAGAGGTTTAACAAAACACAATTTAATTATTTTTATTGATAGTCAAATTTTGAATTAATCTTCAAAACTTTCAACAACGGATCTCTTGGTTCTCGCATCGATGAAGAACGCAGCGAAATGCGATAAGTAATATGAATTGCAGATTTTCGTGAATCATCGAATCTTTGAACGCACATTGCGCCCTCTGGTATTCCAGAGGGCATGCCTGTTTGAGCGTCATTTCTCTCTCAAACCCCCGGGTTTGGTATTGAGTGATACTCTTAGTCGAACTAGGCGTTTGCTTGAAAAGTATTGGCATGGGTAGTACTGGATAGTGCTGTCGACCTCTCAATGTATTAGGTTTATCCAACTCGTTGAATGGTGTGGCGGGATATTTCTGGTATTGTTGGCCCGGCCTTACAACAACCAAACAAGTTTGACCTCAAATCAGG

> AMf4

TTTATTACCCTTGTCTTTTGCGCACTTGTTGTTTCCTGGGCGGGTTCGCCCGCCACCAGGACCACATGATAAACCTTTTTTATGCAGTTGCAATCAGCGTCAGTACAACAAATGTAAATCATTTACAACTTTCAACAACGGATCTCTTGGTTCTGGCATCGATGAAGAACGCAGCGAAATGCGATACGTAGTGTGAATTGCAGAATTCAGTGAATCATCGAATCTTTGAACGCACATTGCGCCCTTTGGTATTCCAAAGGGCATGCCTGTTCGAGCGTCATTTGTACCCTCAAGCTTTGCTTGGTGTTGGGCGTTTTGTCTTTGGTTGCCAAAGACTCGCCTTAAAACGATTGGCAGCCGGCCTACTGGTTTCGCAGCGCAGCACATTTTTGCGCTTGCAATCAGCAAAAGAGGACGGCACTCCATCA AGACTCTTTATCACTTTTGACCTCGGATCAGGTAGGGATACCCG

> AMr10

ACCTTAGTTGCTTCGGCGGGCCCGCCATTNATGGCCGCCGGGGGCTCTCAGCCCCGGGCCCGCGCCCGCCGGAGACACCACGAACTCTGTCTGATCTAGTGAAGTCTGAGTTGATTGTATCGCAATCAGTTAAAACTTTCAACAATGGATCTCTTGGTTCCGGCATCGATGAAGAACGCAGCGAAATGCGATAACTAGTGTGAATTGCAGAATTCCGTGAATCATCGAGTCTTTGAACGCACATTGCGCCCCCTGGTATTCCGGGGGGCATGCCTGTCCGAGCGTCATTGCTGCCCATCAAGCACGGCTTGTGTGTTGGGTCGTCGTCCCCTCTCCGGGGGGGACGGGCCCCAAAGGCAGCGGCGGCACCGCGTCCGATCCTCGAGCGTATGGGGCTTTGTCACCCGCTCTGTAGGCCCGGCCGGCGCTTGCCGAACGCAAATCAATCTTTTCCAGGTTGACCTCGGATCAGGTAGGGATACCCGCTGAACTTAAGCATAT

> AMs9

TTCGGCGGGCCCGCCGTCTTCGGACGGCCGCCGGGGAGGCCTCCGCGCCCCCGGGCCCGCGCCCGCCGAAGACCACAACATGAACTCTGTTCTGAAGTTTTGCAGTCTGAGTTGATTATCATAATCAGTTAAAACTTTCAACAACGGATCTCTTGGTTCCGGCATCGATGAAGAACGCAGCGAAATGCGATAACTAATGTGAATTGCAGAATTCAGTGAATCATCGAGTCTTTGAACGCACATTGCGCCCCCTGGTATTCCGGGGGGCATGCCTGTCCGAGCGTCATTGCTGCCCTCAAGCACGGCTTGTGTGTTGGGCCCCCGTCCCCGCCTCACCGCGGGGACGGGCCCGAAAGGCAGCGGCGGCACCGCGTCCGGTCCTCGAGCGTATGGGGCTTTGTCACCCGCTCTTGTAGGCCCGGCCGGCGCCTGTCGACACCAACCCCAATTTTTCTAAGGTTGACCTCGGATC

> AMsb23

TTTGGCGGGCCCACCGGGGCCACCTGGTCGCCGGGGGACGCACGTCTCCGGGCCCGCGCCCGCCGAAGCGCTCTGTGAACCCTGATGAAGATGGGCTGTCTGAGTACTGTGAAAATTGTCAAAACTTTCAACAATGGATCTCTTGGTTCCGGCATCGATGAAGAACGCAGCGAAATGCGATAAGTAATGTGAATTGCAGAATTCCGTGAATCATCGAATCTTTGAACGCACATTGCGCCCCCTGGCATTCCGGGGGGCATGCCTGTCCGAGCGTCATTTCTGCCCTCAAGCACGGCTTGTGTGTTGGGTGTGGTCCCCCCGGGGACCTGCCCGAAAGGCAGCGGCGACGTCCGTCTGGTCCTCGAGCGTATGGGGCTCTGTCACTCGCTCGGGAAGGACCTGCGGGGGTTGGTCACCACCATGTTTTACCACGGTTGACCTCGGATCAGGTAGGAGTTACCCGCTGAACTTAAGCATATCAAT

> AMs3

TTGCCTCGGCGGGCCCCGCGcCCgcCGACGGCCCCCCTGAACGCTGTCTGAAGTTGCAGTCTGAGACCTATAACGAAATTAgTTAAAACTTTCAACAACGGATCTCTTGGTTCCGGCATCGATGAAGAACGCAGCGAAATGCGATAACTAATGTGAATTGCAGAATTCAGTGAATCATCGAGTCTTTGAACGCACATTGCGCCCTCTGGTATTCCGGAGGGCATGCCTGTCCGAGCGTCATTGCTGCCCTCAAGCCCGGCTTGTGTGTTGGGCCCCGTCCCCCCCGCCGGGGGGACGGGCCCGAAAGGCAGCGGCGGCACCGCGTCCGGTCCTCGAGcGTATGGGGCTTCNTCACCCGCTCTAGTAGGCCCGGCCGGCGCCNNCCNACCCCCAACCTTTAATTATCTCAGGTTGACCTCGGATCAGGTANGGATACCCGCTGAACTTAANCATA

> AMtf5

ATACCCTGTTGCTTTGGCGGGCCCACCGGGGCCACCCCGGTCGCCGGGGGGCGAACACACGCCCCCGGGCCCGCGCCCGCCAGAGCGCTCTGTGAACCCTTATGAAGATGGACTGTCTGAGTGTCTTGATAATAATCAAAACTTTCAACAATGGATCTCTTGGTTCCGGCATCGATGAAGAACGCAGCGAAATGCGATAAGTAATGTGAATTGCAGAATTCCGTGAATCATCGAATCTTTGAACGCACATTGCGCCCCCTGGCATTCCGGGGGGCATGCCTGTCCGAGCGTCATTTCTGCCCTCAAGCACGGCTTGTGTG

> AMb7

ACCTTANTTGCTTCGGCGGGCCCGCCATTCATGGCCGCCGGGGGCTCTCAGCCCCGGGCCCGCGCCCGCCGGAGACACCACGAACTCTGTCTGATCTAGTGAAGTCTGAGTTGATTGTATCGCAATCAGTTAAAACTTTCAACAATGGATCTCTTGGTTCCGGCATCGATGAAGAACGCAGCGAAATGCGATAACTAGTGTGAATTGCAGAATTCCGTGAATCATCGAGTCTTTGAACGCACATTGCGCCCCCTGGTATTCCGGGGGGCATGCCTGTCCGAGCGTCATTGCTGCCCATCAAGCACGGCTTGTGTGTTGGGTCGTCGTCCCCTCTCCGGGGGGGACGGGCCCCAAAGGCAGCGGCGGCACCGCGTCCGATCCTCGAGCGTATGGGGCTTTGTCACCCGCTCTGTAGGCCCGGCCGGCGCTTGCCGAACGCAAATCAATCTTTTTCCAGGTTGACCTCGGATCAGGTAGGGATACCCGCTGAACTTAAGCATA

> AMrb9

TTCGGCGGGCCCGCCGTCTTCGGACGGCCGCCGGGGAGGCCTCCGCGCCCCCGGGCCCGCGCCCGCCGAAGACCACAACATGAACTCTGTTCTGAAGTTTTGCAGTCTGAGTTGATTATCATAATCAGTTAAAACTTTCAACAACGGATCTCTTGGTTCCGGCATCGATGAAGAACGCAGCGAAATGCGATAACTAATGTGAATTGCAGAATTCAGTGAATCATCGAGTCTTTGAACGCACATTGCGCCCCCTGGTATTCCGGGGGGCATGCCTGTCCGAGCGTCATTGCTGCCCTCAAGCACGGCTTGTGTGTTGGGCCCCCGTCCCCGCCTCACCGCGGGGACGGGCCCGAAAGGCAGCGGCGGCACCGCGTCCGGTCCTCGAGCGTATGGGGCTTTGTCACCCGCTCTTGTAGGCCCGGCCGGCGCCTGTCGACACCAACCCCAATTTTTCTAAGGTTGACCTCGGATCAGGTAGGGATACCCGCT

> AMtw3

GCTTCGGCGGGAATAGACGGCCCCGTGAAACGGGCCGCCCCCGCCAGAGGACCCCTAACTCTGTTTCTATAATGTTTCTTCTGAGTAAAACAAGCAAATAAATTAAAACTTTCAACAACGGATCTCTTGGCTCTGGCATCGATGAAGAACGCAGCGAAATGCGATAAGTAATGTGAATTGCAGAATTCAGTGAATCATCGAATCTTTGAACGCACATTGCGCCCGCCAGTATTCTGGCGGGCATGCCTGTTCGAGCGTCATTACAACCCTCAGGCCCCCGGGCCTGGCGTTGGGGATCGGCGGAGCCCCCCGTGGGCACACGCCGTCCCCCAAATACAGTGGCGGTCCCGCCGCAGCTTCCATCGCGTAGTAGCTAACACCTCGCGACTGGAGAGCGGCGCGGCCACGCCGTAAAACACCCAACTCTTCTGAAGTTGACCTCGAATCANGTAGGAATACCCGCT

> AMf1

TATGTTGCCTCGGCGGGCCCCGCGCCCGCCGACGGCCCCCCTGAACGCTGTCTGAAGTTGCAGTCTGAGACCTATAACGAAATTAGTTAAAACTTTCAACAACGGATCTCTTGGTTCCGGCATCGATGAAGAACGCAGCGAAATGCGATAACTAATGTGAATTGCAGAATTCAGTGAATCATCGAGTCTTTGAACGCACATTGCGCCCTCTGGTATTCCGGAGGGCATGCCTGTCCGAGCGTCATTGCTGCCCTCAAGCCCGGCTTGTGTGTTGGGCCCCGTCCCCCCCGCCGGGGGGACGGGCCCGAAAGGCAGCGGCGGNACCGCGTCCGGTCCTCGAGCGTATGGGGCTTCGTCACCCGCTCTAGTAGGCCCGGCCGGCGCCNGCCGACCCCCAACCTTTAATTATCTCAGGTTGACCTCGGATCAGGTAGGGATACCCGCTGAACTTAAGCATA

> AMr9

TGTTGCTTCGGCGGGCCCGCCGCTTGTCGGCCGCCGGGGGGGCGCCTCTGCCCCCCGGGCCCGTGCCCGCCGGAGACCCCAACACGAACACTGTCTGAAAGCGTGCAGTCTGAGTTGATTGAATGCAATCAGTTAAAACTTTCAACAATGGATCTCTTGGTTCCGGCATCGATGAAGAACGCAGCGAAATGCGATAACTAATGTGAATTGCAGAATTCAGTGAATCATCGAGTCTTTGAACGCACATTGCGCCCCCTGGTATTCCGGGGGGCATGCCTGTCCGAGCGTCATTGCTGCCCTCAAGCCCGGCTTGTGTGTTGGGTCGCCGTCCCCCTCTCCGGGGGGACGGGCCCGAAAGGCAGCGGCGGCACCGCGTCCGATCCTCGAGCGTATGGGGCTTTGTCACATGCTCTGTAGGATTGGCCGGCGCCTGCCGACGTTTTCCAACCATTCTTTCCAGGTTGACCTCGGATCAGGTAGGGATACCCGCTGAACTTAAGCATAT

> AMrb1

TTGTTGCTTCGGCAGGCCCGCCTCACGGcCGCCGGGGGGCTTCTCGCCCCCGGGCCCGCGCCTGCCGGAGACACCTTTGAACGCTGTCTGAAGTTTGCAGTCTGAGCGATTAGCTAAATTAGTTAAAACTTTCAACAACGGATCTCTTGGTTCCGGCATCGATGAAGAACGCAGCGAAATGCGATAATTAATGTGAATTGCAGAATTCAGTGAATCATCGAGTCTTTGAACGCACATTGCGCCCCCTGGTATTCCGGGGGGCATGCCTGTCCGAGCGTCATTGCTGCCCTCAAGCCCGGCTTGTGTGTTGGGCCTCGTCCCCCCTCTGCGGGGGACGGGCCCGAAAGGCAGCGGCGGCACCGTGTCCGGTCCTCNAGCNTATGGGGCTTTGTCACCCGCTCTG

> AMrb11 CAGCCTCCCACCCGTGTTGCCCGAACCTATGTTGCCTCGGCGGGCCCCGCGCCCGCCGACGGCCCCCCTGAACGTGTCTGAAGTTGCAGTCTGAGACCTATAACGAAATTAGTTAAAACTTTCAACAACGGATCTCTTGGTTCCGGCATCGATGAAGAACGCAGCGAAATGCGATAACTAATGTGAATTGCAGAATTCAGTGAATCATCGAGTCTTTGAACGCACATTGCGCCCTC TGGTATTCCGGAGGGCATGCCTGTCCGAGCGTCATTGCTGCCCTCAAG CCCGGCTTGTGTGTTGGGCCCCGTCCCCCCCGCCGGGGGGACGGGCCCGAAAGGCAGCGGCGGCACCGCGTCCGGTCCTCGAGCGTATGGGGCTTCGTCACCCGCTCTAGTAGGCCCGGCCGGCGCCAGCCGACCCCCAACCTTTAATTATCTCAGGTTGACCTCGGATCAGGTAGGGATACCCGCTGAACTTAAGCATATCAATAAAGCGATAAGTACCGAGTGCGGGCCCTCGGGGCCCAACCTCCCCCGTGTTGCCGAACTATGTTGCCTCGGCGGGCCCGCGCGCCGACGGCCCCTGAAGCTGTCTGAAGTTGCAGTCTGAGACCTATAACGAAATTAGTTAAAACTTTCAACAACGGATCTCTTGGTTCGGCAT

**Table S3*.*** Means of ratio of the OD at each time interval versus the OD at 0 min (in %) of endophytic fungi extracts against *K. oxytoca* isolate to evaluate the bacteriolysis activity

| Incubation times (h) | | 0 | 1 | 2 | 4 |
| --- | --- | --- | --- | --- | --- |
| Extract codes | **Concentrations** | **Means of ratio of the OD at each time interval versus the OD at 0 min (in %)** | | | |
| [*F.waltergamsii*](https://www.ncbi.nlm.nih.gov/Taxonomy/Browser/wwwtax.cgi?id=2748161) AMtw3 | **4 MIC** | 100±0.00^a^ | 60.464±0.824^a^ | \| 39.320±0.11^a^ \| \| --- \| \|  \| | 29.056±0.539^a^ |
|  | **2 MIC** | 100±0.001^a^ | 64.469±0.041^a^ | 41.353±0.082^a^ | 33.359±0.041^a^ |
|  | **MIC** | 100±0.000^a^ | 66.600±0.404^a^ | 41.518±0.030^a^ | 34.343±2.052^a^ |
| Aspergillus sp. AMtf15 | **4 MIC** | 100±0.000^a^ | 73.795±0.45^a^ | 60.594±0.744^b^ | 38.106±0.512^a^ |
|  | **2 MIC** | 100±1.003^a^ | 77.887±0.412^a^ | 64.739±0.313^b^ | 45.062±0.022^b^ |
|  | **MIC** | 100±0.002^a^ | 80.858±0.228^a^ | 68.165±0.842^b^ | 46.118±2.923^b^ |
| *P. citrinum* AMf6 | **4 MIC** | 100±0.005^a^ | 78.627±0.365^a^ | 54.369±0.821^b^ | 40.165±0.439^b^ |
|  | **2 MIC** | 100±0.007^a^ | 80.389±0.384^a^ | 56.033±0.914^b^ | 44.561±0.288^b^ |
|  | **MIC** | 100±0.000^a^ | 84.132±0.510^a^ | 56.178±0.379^b^ | 48.079±0.446^b^ |
| *Curvularia* sp AMf4 | **4 MIC** | 100±0.000^a^ | 81.320±0.604^a^ | 64.475±0.544^b^ | 45.102±0.459^b^ |
|  | **2 MIC** | 100±0.000^a^ | 84.118±0.532^a^ | 68.363±0.699^b^ | 45.201±1.882^b^ |
|  | **MIC** | 100±0.006^a^ | 84.528±1.092^a^ | 69.617±0.883^b^ | 47.115±1.749^b^ |
| [*T.annesophieae*](https://www.ncbi.nlm.nih.gov/Taxonomy/Browser/wwwtax.cgi?id=2755112) AMsb23 | **4 MIC** | 100±0.000^a^ | 75.72±0.338 ^a^ | 60.877±0.416^b^ | 36.996±1.143^a^ |
|  | **2 MIC** | 100±0.000^a^ | 80.066±0.695^a^ | 64.620±1.507^b^ | 43.128±1.116^b^ |
|  | **MIC** | 100±0.077^a^ | 86.930±2.400^a^ | 69.161±1.506 ^b^ | 47.894±1.179^b^ |
| Ciprofloxacin | **MIC** | 100±000^a^ | 39.584±1.337^b^ | 32.627±0.639^a^ | 25.016±0.302^a^ |
| NC |  | 100±000^a^ | 100±001^c^ | 100±0.000^c^ | 100±0.000^c^ |

AMf: *A. muricata* fruit, AMtf: *A. muricata* thorn of fruit, AMtw: *A. muricata* twigs, AMsb: *A. muricata* stem bark, NC: Negative control, OD: Optical Density, MIC: Minimum Inhibitory Concentrations. Along the column, values carrying the same letter superscripts are not significantly different (p >0.05) and values carrying different letters are significantly different (P˂0.05).

**Table S4*.*** Means of ratio of the OD at each time interval versus the OD at 0 min (in %) of endophytic fungi extracts against *S. aureus* ATCC 43300 to evaluate the bacteriolysis activity

| Incubation times (h) | | 0 | 1 | 2 | 4 |
| --- | --- | --- | --- | --- | --- |
| Extract codes | **Concentrations** | **Means of ratio of the OD at each time interval versus the OD at 0 min (in %)** | | | |
| [*F.waltergamsii*](https://www.ncbi.nlm.nih.gov/Taxonomy/Browser/wwwtax.cgi?id=2748161) AMtw3 | **4 MIC** | 100±0.00^a^ | 62.992±2.307^a^ | 45.817±5.951^a^ | 31.334±0.251^a^ |
|  | **2 MIC** | 100±0.00^a^ | 64.607±8.720^a^ | 49.362±3.015^a^ | 36.064±2.389^a^ |
|  | **MIC** | 100±0.00^a^ | 66.666±3.943^a^ | 51.338±3.123^a^ | 37.760±0.677^a^ |
| Aspergillus sp. AMtf15 | **4 MIC** | 100±0.00^a^ | 80.437±2.711^b^ | 64.687±8.633^b^ | 45.991±1.665^a^ |
|  | **2 MIC** | 100±0.00^a^ | 80.952±4.792^b^ | 66.611±2.429^b^ | 50.624±1.80^b^ |
|  | **MIC** | 100±0.00^a^ | 87.361±8.814^b^ | 75.939±14.65^b^ | 53.769±4.914^b^ |
| *P. citrinum* AMf6 | **4 MIC** | 100±0.000^a^ | 72.522±2.286^b^ | 60.987±2.711^b^ | 48.980±1.385^b^ |
|  | **2 MIC** | 100±0.000^a^ | 90.926±4.189^b^ | 69.626±2.916^b^ | 52.728±3.243^b^ |
|  | **MIC** | 100±0.000^a^ | 92.277±1.265^b^ | 75.958±0.313^b^ | 56.608±2.154^b^ |
| *Curvularia* sp AMf4 | **4 MIC** | 100±0.001^a^ | 84.620±11.145^b^ | 71.480±4.459^b^ | 50.424±2.940^b^ |
|  | **2 MIC** | 100±0.000^a^ | 89.942±6.103^b^ | 72.996±3.145^b^ | 53.400±0.136^b^ |
|  | **MIC** | 100±0.004^a^ | 91.119±7.469^b^ | 74.212±1.999^b^ | 59.066±0.021^b^ |
| [*T.annesophieae*](https://www.ncbi.nlm.nih.gov/Taxonomy/Browser/wwwtax.cgi?id=2755112) AMsb23 | **4 MIC** | 100±0.000^a^ | 78.500±0.796^b^ | 68.221±0.612^b^ | 43.830±0.064^a^ |
|  | **2 MIC** | 100±0.000^a^ | 83.140±5.985^b^ | 71.806±0.299^b^ | 48.803±2.251^b^ |
|  | **MIC** | 100±0.000^a^ | 99.710±3.786^b^ | 74.421±0.618^b^ | 49.609±1.150^b^ |
| Ciprofloxacin | **MIC** | 100±0.000^a^ | 48.217±4.989^c^ | 39.687±0.481^c^ | 30.875±2.702^a^ |
| NC |  | 100±0.000^a^ | 100±0.000^d^ | 100±0.000^d^ | 100±0.003^c^ |

AMf: *A. muricata* fruit, AMtf: *A. muricata* thorn of fruit, AMtw: *A. muricata* twigs, AMsb: *A. muricata* stem bark, NC: Negative control, OD: Optical Density, MIC: Minimum Inhibitory Concentrations. Along the column, values carrying the same letter superscripts are not significantly different (p >0.05) and values carrying different letters are significantly different (P˂0.05).

**Table S5*.*** Means of growth parameter (OD/450 nm) of *K. oxytoca* treated with endophytic fungi extracts for the outer membrane permeability assay

| Extract codes | Means of growth parameter (OD/450 nm) | | | | | | |
| --- | --- | --- | --- | --- | --- | --- | --- |
| Concentrations | **4MIC** | **2MIC** | **MIC** | **1/2 MIC** | **1/4 MIC** | **1/8 MIC** | **1/16 MIC** |
| [*F.waltergamsii*](https://www.ncbi.nlm.nih.gov/Taxonomy/Browser/wwwtax.cgi?id=2748161) AMtw3 | 0.123 ±0.009^a^ | 0.126±0.003^a^ | 0.424±0.037^b^ | 0.881±0.024^a^ | 0.907±0.037 ^a^ | 0.911±0.06 ^a^ | 0.943±0.710^a^ |
| Aspergillus sp. AMtf15 | 0.122±0.004^a^ | 0.125±0.002^a^ | 0.290±0.006^a^ | 0.815±0.021^a^ | 0.862±0.028 ^a^ | 0.893±0.030^a^ | 0.907±0.042^a^ |
| *P. citrinum* AMf6 | 0.153±0.013^a^ | 0.160±0.005^a^ | 0.179±0.016^a^ | 0.573±0.012^b^ | 0.656±0.012^b^ | 0.914±0.010 ^a^ | 0.938±0.031^a^ |
| *Curvularia* sp AMf4 | 0.160±0.007^a^ | 0.181±0.000^a^ | 0.278±0.077 ^a^ | 0.668±0.032^b^ | 0.944±0.006 ^a^ | 0.966±0.020 ^a^ | 1.074±0.097 ^a^ |
| [*T.annesophieae*](https://www.ncbi.nlm.nih.gov/Taxonomy/Browser/wwwtax.cgi?id=2755112) AMsb23 | 0.159±0.039^a^ | 0.159±0.022^a^ | 0.404±0.114^b^ | 0.846±0.027^a^ | 0.852±0.005^a^ | 0.940±0.045 ^a^ | 0.956±0.012^a^ |
| Ciprofloxacin | 0.137±0.016^a^ | 0.251±0.024^a^ | 0.384±0.069^b^ | 0.559±0.032^b^ | 0.764±0.018 ^a^ | 0.851±0.028^a^ | 1.058±0.028^a^ |

AMf: *A. muricata* fruit, AMtf: *A. muricata* thorn of fruit, AMtw: *A. muricata* twigs, AMsb: *A. muricata* stem bark, OD: Optical Density, MIC: Minimum Inhibitory Concentrations. Along the column, values carrying the same letter superscripts are not significantly different (p >0.05) and values carrying different letters are significantly different (P˂0.05).

**Table S6*.*** Means of growth parameter (OD/450 nm) of *S. aureus* ATCC 43300 treated with endophytic fungi extracts for the outer membrane permeability assay

| Extract codes | Means of growth parameter (OD/450 nm) | | | | | | |
| --- | --- | --- | --- | --- | --- | --- | --- |
| Concentrations | **4MIC** | **2MIC** | **MIC** | **1/2 MIC** | **1/4 MIC** | **1/8 MIC** | **1/16 MIC** |
| [*F.waltergamsii*](https://www.ncbi.nlm.nih.gov/Taxonomy/Browser/wwwtax.cgi?id=2748161) AMtw3 | 0.1240.004±^a^ | 0.193±0.055 ^a^ | 0.428±0.027^a^ | 0.537±0.033 ^a^ | 0.734 ±0.038^a^ | 0.749±0.022 ^a^ | 0.856±0.016^a^ |
| Aspergillus sp. AMtf15 | 0.183±0.024 ^a^ | 0.225±0.000 ^a^ | 0.249±0.013^b^ | 0.370 ±0.039^a^ | 0.428±0.010 ^a^ | 0.543±0.026 ^a^ | 0.630 ±0.047^a^ |
| *P. citrinum* AMf6 | 0.238 ±0.019^a^ | 0.243±0.020 ^a^ | 0.257±0.079 ^b^ | 0.424 ±0.002^a^ | 0.500±0.036^a^ | 0.757±0.003^a^ | 0.770±0.034^a^ |
| *Curvularia* sp AMf4 | 0.157±0.032 ^a^ | 0.165±0.020^a^ | 0.213±0.054 ^b^ | 0.360±0.035 ^a^ | 0.405±0.022 ^a^ | 0.624±0.049^a^ | 0.728±0.017^a^ |
| [*T.annesophieae*](https://www.ncbi.nlm.nih.gov/Taxonomy/Browser/wwwtax.cgi?id=2755112) AMsb23 | 0.159±0.003 ^a^ | 0.164±0.023 ^a^ | 0.172±0.021 ^b^ | 0.349±0.042^a^ | 0.402±0.026^a^ | 0.612±0.028 ^a^ | 0.667±0.033^a^ |
| Ciprofloxacin | 0.165±0.05 ^a^ | 0.173±0.010 ^a^ | 0.243±0.030 ^b^ | 0.380±0.092 ^a^ | 0.553±0.024^a^ | 0.627±0.005^a^ | 0.967±0.009^a^ |

AMtf: *A. muricata* thorn of fruit, AMtw: *A. muricata* twigs, AMsb: *A. muricata* stem bark, OD: Optical Density, MIC: Minimum Inhibitory Concentrations. Along the column, values carrying the same letter superscripts are not significantly different (p >0.05) and values carrying different letters are significantly different (P˂0.05).

**Table S7*.*** Means of optical densities (620nm) at each time interval of *K.oxytoca* isolate treated with endophytic fungi extracts for the time kill kinetics assay

| Incubation time (h) | | 0 | 1 | 2 | 4 | 6 | 8 | 10 | 12 | 24 |
| --- | --- | --- | --- | --- | --- | --- | --- | --- | --- | --- |
| Extract codes | **Concentrations** | **OD (620nm)** | | | | | | | | |
| [*F.waltergamsii*](https://www.ncbi.nlm.nih.gov/Taxonomy/Browser/wwwtax.cgi?id=2748161) AMtw3 | **4 MIC** | 0.218±0.022^a^ | 0.327±0.007 ^a^ | 0.312±0.003 ^a^ | 0.347±0.043 ^a^ | 0.312±0.025 ^a^ | 0.215±0.032^a^ | 0.192±0.008 ^a^ | 0.171±0.001^a^ | 0.153±0.003^a^ |
|  | **2 MIC** | 0.208±0.015^a^ | 0.318±0.006 ^a^ | 0.320±0.004 ^a^ | 0.319±0.010 ^a^ | 0.334±0.018 ^a^ | 0.284±0.000 ^a^ | 0.325±0.012^a^ | 0.3421±0.00^b^ | 0.203±0.035^a^ |
|  | **MIC** | 0.202±0.005^a^ | 0.328±0.003 ^a^ | 0.311±0.003^a^ | 0.302±0.013^a^ | 0.335±0.001^a^ | 0.275±0.003 ^a^ | 0.318±0.007^a^ | 0.331±0.019 ^b^ | 0.185±0.063^a^ |
| Aspergillus sp. AMtf15 | **4 MIC** | 0.263±0.013^a^ | 0.383±0.009^a^ | 0.377±0.011 ^a^ | 0.346±0.020 ^a^ | 0.386±0.011 ^a^ | 0.319±0.017^a^ | 0.359±0.018^a^ | 0.386±0.002 ^b^ | 0.266±0.014 ^a^ |
|  | **2 MIC** | 0.337±0.010 ^a^ | 0.447±0.003^a^ | 0.438±0.001 ^a^ | 0.389±0.008^a^ | 0.449±0.000 ^a^ | 0.371±0.000 ^a^ | 0.404±0.006 ^a^ | 0.429±0.003^b^ | 0.316±0.042^b^ |
|  | **MIC** | 0.318±0.017 ^a^ | 0.339±0.002^a^ | 0.348±0.001 ^a^ | 0.529±0.022^a^ | 0.524±0.011^b^ | 0.503±0.013^b^ | 0.515±0.006^b^ | 0.488±0.004 ^b^ | 0.430±0.018^b^ |
| *P. citrinum* AMf6 | **4 MIC** | 0.238±0.009 ^a^ | 0.378±0.008^a^ | 0.384±0.001 ^a^ | 0.395±0.001^a^ | 0.385±0.008^a^ | 0.328±0.000 ^a^ | 0.360±0.003 ^a^ | 0.364±0.000 ^b^ | 0.276±0.000 ^a^ |
|  | **2 MIC** | 0.283±0.002^a^ | 0.425±0.010^a^ | 0.424±0.009^a^ | 0.396±0.005^a^ | 0.436±0.007^a^ | 0.373±0.003 ^a^ | 0.393±0.003 ^a^ | 0.438±0.008 ^b^ | 0.405±0.005^b^ |
|  | **MIC** | 0.400±0.015 ^a^ | 0.541±0.017^b^ | 0.540±0.000 ^b^ | 0.564±0.003^b^ | 0.545±0.022 ^b^ | 0.452±0.016^b^ | 0.482±0.003^b^ | 0.462±0.003^b^ | 0.447±0.000 ^b^ |
| *Curvularia* sp AMf4 | **4 MIC** | 0.270±0.021 ^a^ | 0.624±0.000 ^b^ | 0.637±0.021 ^b^ | 0.622±0.003^b^ | 0.458±0.040 ^a^ | 0.457±0.001 ^b^ | 0.442±0.002 ^a^ | 0.405±0.000 ^b^ | 0.372±0.000 ^b^ |
|  | **2 MIC** | 0.464±0.006 ^a^ | 0.586±0.011^b^ | 0.552±0.027 ^b^ | 0.557±0.000^b^ | 0.574±0.028^b^ | 0.510±0.011 ^b^ | 0.518±0.004^b^ | 0.553±0.002^b^ | 0.445±0.004 ^b^ |
|  | **MIC** | 0.328±0026^a^ | 0.653±0.044^b^ | 0.908±0.061 ^c^ | 0.939±0.010^c^ | 0.908±0.02^c^ | 0.795±0.038^c^ | 0.792±0.020^c^ | 0.810±0.016^c^ | 0.660±0.025^c^ |
| [*T.annesophieae*](https://www.ncbi.nlm.nih.gov/Taxonomy/Browser/wwwtax.cgi?id=2755112) AMsb23 | **4 MIC** | 0.269±0.015 ^a^ | 0.378±0.007^a^ | 0.356±0.012^a^ | 0.370±0.004 ^a^ | 0.355±0.003^a^ | 0.369±0.010 ^a^ | 0.368±0.009 | 0.391±0.004 ^b^ | 0.269±0.020 ^a^ |
|  | **2 MIC** | 0.264±0.032 ^a^ | 0.350±0.013 ^a^ | 0.328±0.003 ^a^ | 0.312±0.002 ^a^ | 0.310±0.061 ^a^ | 0.288±0.004 ^a^ | 0.322±0.040 ^a^ | 0.349±0.023 ^b^ | 0.217±0.020 ^a^ |
|  | **MIC** | 0.246±0.020 ^a^ | 0.335±0.002 ^a^ | 0.311±0.015^a^ | 0.298±0.001 ^a^ | 0.278±0.104^a^ | 0.260±0.018 ^a^ | 0.306±0.049 ^a^ | 0.328±0.003^b^ | 0.209±0.063^a^ |
| Ciprofloxacin | **MIC** | 0.224±0.010 ^a^ | 0.331±0.006 ^a^ | 0.330±0.007^a^ | 0.289±0.002 ^a^ | 0.222± ^a^0.008 | 0.237±0.005 ^a^ | 0.333±0.015 ^a^ | 0.348±0.001 ^b^ | 0.254±0.007^a^ |
| NC |  | 0.238±0.018 ^a^ | 0.336±0.004^a^ | 0.334±0.006 ^a^ | 0.335±0.013 ^a^ | 0.666±0.014^b^ | 0.852±0.004^c^ | 1.160±0.000^d^ | 1.164±0.003^d^ | 1.190±0.012^c^ |

AMf: *A. muricata* fruit, AMtf: *A. muricata* thorn of fruit, AMtw: *A. muricata* twigs, AMsb: *A. muricata* stem bark, OD: Optical Density, MIC: Minimum Inhibitory Concentrations. Along the column, values carrying the same letter superscripts are not significantly different (p >0.05) and values carrying different letters are significantly different (P˂0.05).

**Table S8*.*** Means of optical densities (620nm) at each time interval of *S. aureus* ATCC 43300 treated with endophytic fungi extracts for the time kill kinetics assay

| Incubation time (h) | | 0 | 1 | 2 | 4 | 6 | 8 | 10 | 12 | 24 |
| --- | --- | --- | --- | --- | --- | --- | --- | --- | --- | --- |
| Extracts codes | **Concentrations** | **Means of OD (620nm)** | | | | | | | | |
| [*F.waltergamsii*](https://www.ncbi.nlm.nih.gov/Taxonomy/Browser/wwwtax.cgi?id=2748161) AMtw3 | **4 MIC** | 0.350±0.061 ^a^ | 0.346±0.015 ^a^ | 0.347±0.012 ^a^ | 0.346±0.004 ^a^ | 0.336±0.015 ^a^ | 0.351±0.019 ^a^ | 0.321±0.002 ^a^ | 0.319±0.007 ^a^ | 0.281±0.000 ^a^ |
|  | **2 MIC** | 0.303±0.002 ^a^ | 0.353±0.002 ^a^ | 0.340±0.005 ^a^ | 0.331±0.009 ^a^ | 0.316±0.018 ^a^ | 0.328±0.005 ^a^ | 0.2496±0.04^a^ | 0.288±0.005 ^a^ | 0.262±0.003 ^a^ |
|  | **MIC** | 0.402±0.129 ^a^ | 0.448±0.019 ^a^ | 0.488±0.005 ^a^ | 0.461±0.020 ^a^ | 0.411±0.057 ^a^ | 0.424±0.045 ^a^ | 0.3852±0.03^a^ | 0.342±0.043 ^a^ | 0.305±0.005 ^a^ |
| Aspergillus sp. AMtf15 | **4 MIC** | 0.342±0.013 ^a^ | 0.383±0.012 ^a^ | 0.386±0.028 ^a^ | 0.380±0.009 ^a^ | 0.321±0.013 ^a^ | 0.347±0.007 ^a^ | 0.3423±0.00^a^ | 0.328±0.000 ^a^ | 0.305±0.003 ^a^ |
|  | **2 MIC** | 0.384±0.027 ^a^ | 0.436±0.032 ^a^ | 0.430±0.032 ^a^ | 0.413±0.018 ^a^ | 0.382±0.022 ^a^ | 0.394±0.015 ^a^ | 0.375±0.004 ^a^ | 0.368±0.004 ^a^ | 0.355±0.001 ^a^ |
|  | **MIC** | 0.329±0.011 ^a^ | 0.634±0.007 ^b^ | 0.644±0.016 ^b^ | 0.649±0.002 ^b^ | 0.554±0.029 ^b^ | 0.564±0.028 ^b^ | 0.528±0.028 ^b^ | 0.507±0.018 ^b^ | 0.48±0.012 ^a^ |
| *P. citrinum* AMf6 | **4 MIC** | 0.324±0.002 ^a^ | 0.481±0.004 ^a^ | 0.378±0.016 ^a^ | 0.381±0.017 ^a^ | 0.356±0.055 ^a^ | 0.331±0.008 ^a^ | 0.3038±0.01^a^ | 0.295±0.014 ^a^ | 0.267±0.000 ^a^ |
|  | **2 MIC** | 0.361±0.019 ^a^ | 0.440±0.022 ^a^ | 0.433±0.018 ^a^ | 0.412±0.004 ^a^ | 0.467±0.017 ^b^ | 0.377±0.013 ^a^ | 0.343±0.005 ^a^ | 0.331±0.007 ^a^ | 0.307±0.003 ^a^ |
|  | **MIC** | 0.348±0.048 ^a^ | 0.644±0.033 ^b^ | 0.652±0.440 ^b^ | 0.648±0.063 ^b^ | 0.572±0.002 ^b^ | 0.562±0.023 ^b^ | 0.5049±0.00^a^ | 0.490±0.013 ^a^ | 0.478±0.005 ^a^ |
| *Curvularia* sp AMf4 | **4 MIC** | 0.315±0.012 ^a^ | 0.427±0.032 ^a^ | 0.462±0.017 ^a^ | 0.427±0.003 ^a^ | 0.417±0.000 ^a^ | 0.425±0.030 ^a^ | 0.395±0.001 ^a^ | 0.365±0.005 ^a^ | 0.347±0.000 ^a^ |
|  | **2 MIC** | 0.354±0.035 ^a^ | 0.561±0.024 ^b^ | 0.577±0.028 ^b^ | 0.566±0.027 ^b^ | 0.553±0.036 ^b^ | 0.569±0.045 ^a^ | 0.521±0.003^a^ | 0.505±0.034 ^a^ | 0.467±0.002 ^a^ |
|  | **MIC** | 0.347±0.008 | 0.849±0.056 ^c^ | 0.900±0.065^c^ | 0.910±0.056 ^c^ | 0.795±0.025 ^c^ | 0.793±0.039 ^b^ | 0.729±0.046 ^b^ | 0.710±0.046 ^b^ | 0.668±0.013 ^b^ |
| [*T.annesophieae*](https://www.ncbi.nlm.nih.gov/Taxonomy/Browser/wwwtax.cgi?id=2755112) AMsb23 | **4 MIC** | 0.331±0.002 ^a^ | 0.336±0.010 ^a^ | 0.355±0.015 ^a^ | 0.451±0.003 ^a^ | 0.298±0.015 ^a^ | 0.307±0.094 ^a^ | 0.280±0.071 ^a^ | 0.291±0.073 ^a^ | 0.224±0.002 ^a^ |
|  | **2 MIC** | 0.335±0.001 ^a^ | 0.353±0.006 ^a^ | 0.352±0.015 ^a^ | 0.454±0.006 ^b^ | 0.297±0.003 ^a^ | 0.310±0.079 ^a^ | 0.239±0.007 ^a^ | 0.320±0.001 ^a^ | 0.265±0.044 ^a^ |
|  | **MIC** | 0.311±0.003^a^ | 0.379±0.023^a^ | 0.390±0.016^a^ | 0.427±0.028^a^ | 0.297±0.041^a^ | 0.329±0.067^a^ | 0.253±0.018^a^ | 0.305±0.058^a^ | 0.265±0.037^a^ |
| Ciprofloxacin | **MIC** | 0.326±0.002^a^ | 0.358±0.000^a^ | 0.345±0.011^a^ | 0.341±0.006^a^ | 0.286±0.011^a^ | 0.255±0.116^a^ | 0.246±0.016^a^ | 0.260±0.022^a^ | 0.224±0.002^a^ |
| NC |  | 0.310±0.000 ^a^ | 0.358±0.002^a^ | 0.384±0.005^a^ | 0.566±0.045^b^ | 0.966±0.003^c^ | 1.166±0.045^c^ | 1.415±0.034^c^ | 1.445±0.000^c^ | 1.668±0.000 ^c^ |

AMf: *A. muricata* fruit, AMtf: *A. muricata* thorn of fruit, AMtw: *A. muricata* twigs, AMsb: *A. muricata* stem bark, OD: Optical Density, NC: Negative Control, MIC: Minimum Inhibitory Concentrations. Along the column, values carrying the same letter superscripts are not significantly different (p >0.05) and values carrying different letters are significantly different (P˂0.05)

**Table S9*.*** Reduction factors of *S. aureus* ATCC 43300 and *K. oxytoca isolate* population by endophytic extracts after 24 hours of incubation

|  | Reduction of bacteria population after 24 hours | | | | | |
| --- | --- | --- | --- | --- | --- | --- |
| Strains | ***K. oxytoca*** | | | ***S. aureus* ATCC 43300** | | |
| Fungal extracts | **4MIC** | **2MIC** | **MIC** | **4MIC** | **2MIC** | **MIC** |
| [*F.waltergamsii*](https://www.ncbi.nlm.nih.gov/Taxonomy/Browser/wwwtax.cgi?id=2748161) AMtw3 | 7.76^a^ | 6.14^a^ | 5.80^a^ | 5.92^a^ | 4.53^a^ | 3.90^a^ |
| Aspergillus sp. AMtf15 | 4.47^b^ | 3.76^b^ | 2.76^b^ | 5.46^a^ | 3.35^b^ | 2.44^b^ |
| *P. citrinum* AMf6 | 4.30^b^ | 2.93^c^ | 2.66^b^ | 6.24^b^ | 3.87^b^ | 2.49^b^ |
| *Curvularia* sp AMf4 | 3.19^c^ | 2.67^c^ | 1.80^c^ | 4.79^c^ | 2.54^c^ | 1.78^c^ |
| [*T.annesophieae*](https://www.ncbi.nlm.nih.gov/Taxonomy/Browser/wwwtax.cgi?id=2755112) AMsb23 | 6.45^d^ | 5.47^d^ | 4.42^d^ | 7.42^d^ | 4.56^d^ | 4.49^d^ |
| Ciprofloxacin | NA | NA | 4.67^d^ | NA | NA | 7.11^e^ |

AMr: *A. muricata* root, AMf: *A. muricata* fruit, AMtf: *A. muricata* thorn of fruit, AMtw: *A. muricata* twigs, AMsb: *A. muricata* stem bark. Along the column, values carrying the same letter superscripts are not significantly different (p >0.05) and values carrying different letters are significantly different (P˂0.05).

**Table S10*.*** Amount of biofilm materialised by the mean of relative fluorescence units on *S. aureus* ATCC 43300 and *K. oxytoca isolate* by endophytic extracts after 48 hours of incubation

| Strains | Means of relative fluorescence units |
| --- | --- |
| *K. oxytoca* | 13614.5 ±29.760^a^ |
| *S. aureus* | 12665.75 ±173.240^a^ |

Along the column, values carrying the same letter superscripts are not significantly different (p >0.05).
